# People with hip osteoarthritis have reduced quadriceps voluntary activation and altered motor cortex function

**DOI:** 10.1016/j.smhs.2024.09.005

**Published:** 2024-09-20

**Authors:** Myles C. Murphy, Molly Coventry, Janet L. Taylor, Ebonie K. Rio, Andrea B. Mosler, Jackie L. Whittaker, Christopher Latella

**Affiliations:** a Nutrition and Heath Innovation Research Institute, School of Medical and Health Sciences, Edith Cowan University, Joondalup, Western Australia, Australia; b School of Health Sciences, The University of Notre Dame Australia, Fremantle, Western Australia, Australia; c School of Medical and Health Sciences, Edith Cowan University, Joondalup, Western Australia, Australia; d Neuroscience Research Australia, Randwick, New South Wales, Australia; e La Trobe Sport and Exercise Medicine Research Centre, La Trobe University, Bundoora, Victoria, Australia; f The Australian Ballet, Melbourne, Victoria, Australia; g The Victorian Institute of Sport, Melbourne, Victoria, Australia; h Department of Physical Therapy, Faculty of Medicine, University of British Columbia, Vancouver, Canada; i Centre for Aging SMART, University of British Columbia, Vancouver, Canada; j Arthritis Research Canada, Vancouver, Canada; k Neurophysiology Research Laboratory, School of Medical and Health Sciences, Edith Cowan University, Joondalup, Western Australia, Australia

**Keywords:** Muscle inhibition, Rehabilitation, Arthritis, Hip pain, Physiotherapy

## Abstract

**Aims:**

Compare quadriceps voluntary activation, corticospinal and intracortical excitability between people with and without hip osteoarthritis (OA). Exploratory objectives include quantifying the association of corticospinal/intracortical excitability with voluntary activation, corticospinal/intracortical excitability with hip related pain, and motor threshold with motor cortex inhibition and facilitation.

**Methods:**

Case-control study including participants with clinically and radiologically confirmed hip OA and non-OA controls. Quadriceps voluntary activation was assessed using twitch interpolation via femoral nerve stimulation. Single- and paired-pulse transcranial magnetic stimulation over the motor cortex assessed resting motor threshold (RMT), active motor threshold (AMT), short-interval intracortical inhibition (SICI), intracortical facilitation (ICF) and silent period. Generalized linear models assessed outcomes (*p* ​< ​0.05).

**Results:**

We included 17 hip OA (76% female) and 24 controls (92% female) with a mean (standard deviation) age of 58.7 (7.9) years. Compared to controls, people with hip OA had reduced quadriceps voluntary activation (*β* ​= ​-5.29, 95% confidence intervals [*CI*], -0.79–-9.79) and increased ICF (*β* ​= ​0.22, 95%*CI*, 0.01–0.43). People with hip OA did not differ from controls in RMT (*β* ​= ​−4.76, 95%*CI*, −14.08–4.56), AMT (*β* ​= ​−2.13, 95%*CI*, −7.12-2.86), SICI (*β* ​= ​−0.02, 95%*CI*, −0.15-0.006) or silent period (*β=* 8.72, 95%*CI,* −24.75–42.20). More facilitation was associated with increased hip pain (*β=* 24.55, 95%*CI,* 6.93–42.18), and more inhibition was associated with less voluntary activation (*β=* 10.50, 95%*CI,* 2.00–18.99).

**Conclusion:**

People with hip OA demonstrate reduced quadriceps voluntary activation and complex changes in motor cortex excitability compared to controls. These findings suggest that hip OA can alter quadriceps neuromuscular function (facilitation associated with pain, inhibition associated with activation), thus having implications for rehabilitation.

## Abbreviations

OA: Osteoarthritis
*SMD*: Standardised Mean Difference
TMS: Transcranial Magnetic Stimulation
ICF: Intracortical Facilitation
SP: Silent Period
ACLR: Anterior Cruciate Ligament Reconstruction
VM: Vastus Medialis
VL: Vastus Lateralis
RF: Rectus Femoris
*ICC*: Intraclass Correlation Coefficient
Mmax: Maximal Compound Action Potential
mV: millivolt
RMT: Resting Motor Threshold
AMT: Active Motor Threshold
MVIC: Maximal Voluntary Isometric Contraction
MSO: Maximal Stimulator Output
MEP: Motor Evoked Potential
EMG: Electromyography
SICI: Short-Interval Intracortical Inhibition
HAGOS: Hip and Groin Outcome Score
NRS-P: 11-Point Numerical Rating Scale of Pain
GAD-7: Generalized Anxiety Screen
PHQ-9: Patient Health Questionnaire
DASS: Depression, Anxiety and Stress Scale
95%*CI*: 95​% Confidence Intervals
*SD*: Standard Deviation

## 1 Introduction

Osteoarthritis (OA) results in substantial personal burden (higher years lived with disability than type 2 diabetes or stroke^1^), causes substantial time-loss from work^2^^,^ and is associated with a high economic cost.^3^ Further, people with OA have substantial impairments in muscle activation and function.^4^

The proportion of muscle fibers activated when performing a maximal active contraction is termed voluntary activation. In people with knee OA, this level of recruitment is markedly reduced.^4^ Our meta-analysis of people with knee OA demonstrated a large effect for reduced quadriceps voluntary activation compared to controls without OA (Standardised Mean Difference; SMD [95%*CI*] ​= ​−0.84 [−1.12 to −0.56]),^4^ reductions that predict quadriceps strength.^5^ Alterations in voluntary activation are potential drivers of the pain and physical impairments seen in OA.^6^^,^^7^

Transcranial magnetic stimulation (TMS) over the motor cortex is used to assess the function of the motor pathway and cortex itself.^8^ Specifically, single-pulse TMS can assess the excitability of the corticospinal pathway, while paired-pulse TMS can assess intracortical inhibitory and excitatory circuitry. In knee OA, an increase in intracortical facilitation (ICF) has been associated with both increased,^6^ and decreased pain scores.^9^ Another measure of inhibitory motor function is the silent period (SP), reflecting both spinal and cortical mediated inhibition. The SP duration has been associated with knee OA pain.^10^

Knee OA is not the only lower-limb condition associated with altered motor function. Following anterior cruciate ligament reconstruction (ACLR) quadriceps strength and voluntary activation are impaired,^11^ which may contribute to recalcitrant dysfunction and progression to knee OA.^12^^,^^13^ A recent meta-analysis assessing voluntary activation deficits demonstrated a large effect (*SMD* [95%*CI*] ​= ​−0.84 [−1.18 to −0.50]) for reduced quadriceps voluntary activation in ACLR populations versus controls,^11^ which is almost identical to the effect size observed with knee OA.^4^ This alteration in motor function, and in particular the inability to generate strong contractions (poor voluntary activation) may minimize the tolerance to, and effect of exercise-based interventions.^14^ Thus, assessment of voluntary activation may help identify non-responders to exercise-based rehabilitation, better identify neuromuscular impairment, and inform the design of more targeted exercise-based interventions.

Despite a strong body of evidence about the effects of knee OA and ACLR on quadriceps voluntary activation and cortical function, there is a paucity of evidence about quadriceps voluntary activation and motor function in people with hip OA.

The primary objective of this study was to compare quadriceps voluntary activation, corticospinal excitability, and intracortical excitability between people with and without hip OA. Exploratory objectives to quantify the association of: i) corticospinal/intracortical excitability with voluntary activation, ii) corticospinal/intracortical excitability with hip-related pain, and iii) motor threshold with motor cortex inhibition and facilitation.

We hypothesized that people with hip OA would display impaired quadriceps voluntary activation compared to controls. We also hypothesized that intracortical excitability (inhibition and facilitation) and silent periods would differ from controls. We expect to observe an association of self-reported pain intensity with voluntary activation and motor cortex function. Finally, we expected an association of motor cortex excitability with intracortical inhibition and facilitation.

## 2 Methods

### 2.1 Study design

We performed a cross-sectional study between July 2023 and March 2024. Self-reported data were obtained using an online, electronic Qualtrics survey that was completed within 48 ​hours (h) of the physical assessment. Physical and neurophysiological assessments were performed in the Edith Cowan University Neurophysiology Research Laboratory.

### 2.2 Study protocol

Our study was designed and is reported, in accordance with the STROBE Statement for Cross-sectional Studies.^15^

### 2.3 Ethical approval

This research was reviewed and received approval by the Edith Cowan University Human Research Ethics Committee (2023-04080-MURPHY). The research was implemented in accordance with the Declaration of Helsinki. All participants provided informed, electronic consent.

### 2.4 Participants

We included a convenience sample of physically active participants between 40 and 70 years of age with and without hip OA who did not experience morning stiffness (in any body region) lasting > 30 ​minutes (min). Participants were excluded if they were contraindicated for TMS as outlined in Appendix A.^16^ Hip OA participants were required to have a clinical and radiological diagnosis of hip OA, which could be unilateral or bilateral. A diagnosis of hip OA was accepted when the participant reported activity-related hip and/or groin pain (using the validated Hip and Groin Outcome Score)^17^^,^^18^ and either osteophytic change (bony spurring) or joint space narrowing on X-ray (Kellgren Lawrence Grade of > 2).^19^ In addition to the contraindications for TMS, participants were excluded if they had been diagnosed with an inflammatory polyarthropathy. Participants with unilateral and bilateral hip OA were included, with the most painful side used for reporting. Controls were required to be free of any confounding current lower-limb injuries or other illnesses.

### 2.5 Recruitment

Participants were recruited via clinical networks (e.g., general practitioners), social media (e.g., LinkedIn), governing bodies (e.g., Western Australian branch of the Australian Physiotherapy Association), Edith Cowan University School of Medical and Health Sciences Research Register and word-of-mouth.

### 2.6 Variables

#### 2.6.1 Demographic data

Age (years), sex (female; male; intersex), ethnicity, education level (less than high school degree; high school graduate; bachelor's degree; master's degree; doctoral degree), work status (full-time; part-time; student; retired; other) and socioeconomic status via household income (less than AUD 30 000; AUD 30 000 – $49 999; AUD 50 000 - $79 999; AUD 80 000 - $99 999; AUD 100 000 - $149 999; AUD 150 000 - $199 999; more than AUD 200 000) were reported online via Qualtrics.

#### 2.6.2 Quadriceps strength and muscle activity

**Fig. 1:**
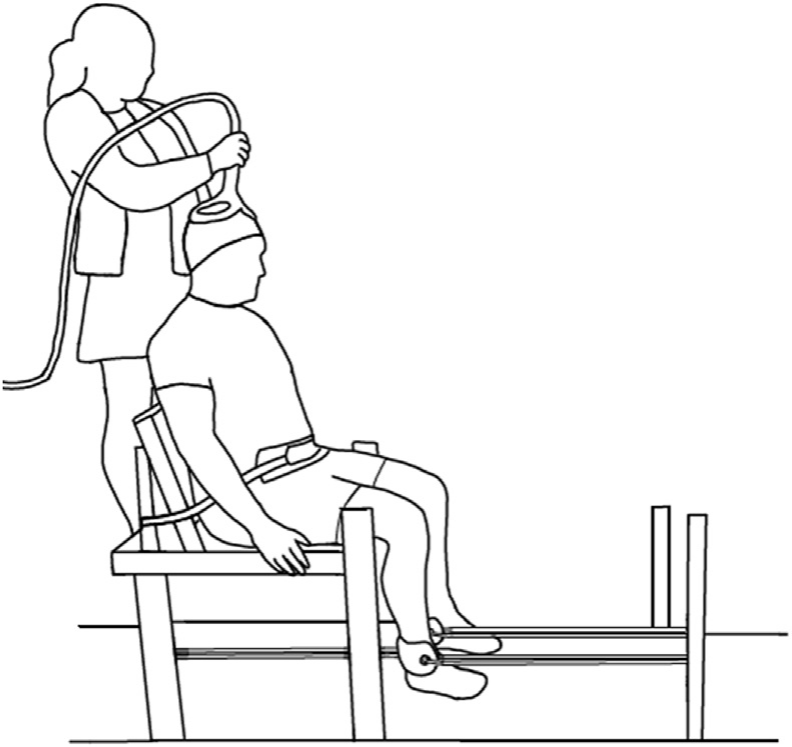
Participant positioning for transcranial magnetic stimulation, femoral nerve stimulation, and quadriceps strength testing.

The assessment of quadriceps strength and activity of the vastus medialis (VM), vastus lateralis (VL), and rectus femoris (RF) muscles are described in Table 1 and Fig. 1.

**Table 1:** Detailed description of neurophysiological assessment procedures.

Outcome	Procedure
Quadriceps Muscle Strength	Participants performed three maximal voluntary isometric contractions of the quadriceps at 90 degrees of knee flexion after performing warm-up trials at 50%, 75% and 90%. Each trial lasted 2–5 ​s with a minimum of 60-s rest between trials. The maximal value of the three trials was recorded as the maximal voluntary isometric contraction. All quadriceps maximal voluntary isometric contractions were performed in a custom-built chair designed to measure muscle forces, with an inline force transducer attached to a rope and cuff that secured to the ankle (Fig. 1).^32^ The leg was secured to an in-line force transducer (UU-K100 100 ​kg, Load cell, Australia) via a Velcro strap 2 ​cm above the ankle and rope attached to an immovable bar behind the leg. Another Velcro strap and rope attached to an anterior immovable bar was used to suspend the leg at the correct angle.
Quadriceps Muscle Activity	Quadriceps muscle bellies were determined based on surface electromyography for non-invasive assessment of muscles guidelines.^33^ The skin over the target muscles was first cleaned with an alcohol wipe and electrode placement marked. Electromyography was recorded via surface electrodes for each of the VM, VL, RF muscles of the tested leg using a pseudomonopolar configuration. Specifically, electrodes were placed on the muscle bellies of RF, VM and VL at 50%, 80% and 66%, respectively, of the distance from the anterior superior iliac spine to patella with additional electrodes ∼50 ​mm distal to each point in line with the muscle fibres. A ground electrode (3M Universal Electrosurgical Pad) was placed over the tibial tubercle. Electromyography signals were amplified and filtered ( ​× ​100; 16-1 000 ​Hz; CED 1902 amplifier, Cambridge Electronic Design, Cambridge, United Kingdom) and then digitised (sampling rate 2 000 ​Hz) using a computer interface (CED 1401 and Spike2 software, Cambridge Electronic Design).
Quadriceps Voluntary Activation	To determine quadriceps voluntary activation, the femoral nerve was stimulated with a brief, electrical stimulus (200-ms pulse duration) using a constant current stimulator (Digitimer DS7A, Letchworth Garden City, United Kingdom) via self-adhesive electrodes on the skin (Kendall ECG electrodes). The anode was placed over the gluteus medius and the optimal stimulation site for the cathode was first determined for each participant using a custom hand-held electrode. The site that elicited the greatest twitch response with a stimulus intensity of ∼30–50 milliamps was then marked before the cathode electrode was placed over the femoral nerve at the level of the inguinal crease. Single stimuli were delivered with steps of increasing intensity until maximal twitch force was evoked from the quadriceps. The stimulation intensity for voluntary activation trials was set at 120% of this value for each participant to ensure a supramaximal stimulus was delivered. During voluntary activation trials, participants were instructed to perform a maximal voluntary isometric contraction, during which a stimulus was given, then to relax completely, with a second stimulus delivered 2–4 ​s after relaxation was achieved.
Transcranial magnetic Stimulation	A double cone coil (11 ​cm outside diameter, Magstim) attached to a magnetic stimulator (Bistim2, Magstim, Whitland, United Kingdom) was used to assess single- (one stimulator discharged) and paired-pulse (two stimulators discharged at pre-specified interstimulus intervals and intensities) responses. The coil was held over the motor cortex contralateral to the participant's test limb, with the coil held so that a posterior-anterior current was induced. The same researcher performed all transcranial magnetic stimulation assessments for consistency. Initially, the coil was moved to identify the ‘hot spot’, which was the location that evoked the largest motor evoked potential in the RF with the muscle at rest or during a 10% maximal voluntary isometric contraction. This position was marked on a swim cap worn by the participant throughout their assessment session, enabling consistent placement of the coil. Single- and paired-pulse motor evoked potential sizes were quantified by measuring the peak-to-peak amplitude (millivolts) of the signal. For each participant, motor evoked potentials were averaged, and single-pulse motor evoked potential amplitudes were expressed as a percentage of Maximal Compound Action Potential. Short-interval intracortical inhibition and intracortical facilitation were expressed as a ratio (amplitude of conditioned motor evoked potential/amplitude of unconditioned motor evoked potential).

Legend: VM ​= ​vastus medialis, VL ​= ​vastus lateralis, RF ​= ​rectus femoris.

#### 2.6.3 Quadriceps voluntary activation

A detailed description of the quadriceps voluntary activation procedure is located in Table 1. Participants performed five trials, with 60-second (s) rests between efforts. For each trial, voluntary activation was quantified offline: voluntary activation (%) was calculated as (1 – superimposed twitch amplitude/resting twitch amplitude) × 100.^20^ The highest activation of the five trials was used for analyses. This technique has excellent reliability (intraclass correlation coefficient [*ICC*] ​= ​0.93) in people with OA.^21^ Maximal muscle compound action potentials - M-waves (Mmax) for each muscle were calculated as the mean peak-to-peak amplitudes (mV) of the EMG potentials recorded across the five trials of the resting twitch (mV).

#### 2.6.4 Corticospinal and intracortical responses

A detailed description of the TMS procedure is located in Table 1. Single-pulse TMS was then delivered to determine each participant's resting and active motor thresholds (RMT and AMT, respectively). For RMT, participants remained relaxed, and for AMT trials the participant was instructed to hold a steady 10% maximal voluntary isometric contraction (MVIC) while a stimulus was delivered. Participants received visual feedback of the quadriceps force signal and a force target on a computer screen. RMT was determined to be the lowest level (percentage) of maximal stimulator output (MSO) to elicit 3/6 Motor Evoked Potentials (MEPs) with an amplitude > 0.05 ​mV in the RF. Similarly, the AMT was the lowest level of MSO to elicit 3/6 RF MEPs that were visually larger than background electromyography (EMG) during a 10% quadriceps MVIC. The AMT value was also used to set stimulator output intensities for subsequent single- and paired-pulse assessments. The conditioning stimulus and test stimulus intensities were set at 80% and 120% of AMT respectively. Single-pulse test stimuli were used to evoke MEPs and an SP, while paired-pulse trials were used to test short-interval intracortical inhibition (SICI) with a conditioning-test interstimulus interval of 3 ​ms and ICF with an interstimulus interval of 12 ​ms. Each participant received a total of 10 × MEP 120%, 10 × SICI, and 10 × ICF stimuli. These were randomized into 5 blocks, each containing 6 stimuli. Each stimulus block was delivered during a 10% quadriceps MVIC lasting ∼30 ​s. Stimuli were delivered each 5 ​s and the participant was instructed to maintain force output at 10% MVIC after each stimulus.

#### 2.6.5 Hip-related pain and disability

The Hip and Groin Outcome Score (HAGOS) is a valid and reliable patient-reported outcome measure and was used to quantify the domains of: symptoms and stiffness; pain; physical function, daily living; function, sports and recreational activities; participation in physical activities; quality of life.^17^^,^^18^ The HAGOS is internationally recommended to evaluate hip-related pain and function in young to middle-aged active people, thus suited to our cohort.^17^ For simplicity scores were inverted so each of these domains is scored on a separate sub-scale between 0% and 100%, with a higher score representing worse pain, and disability.^17^^,^^18^

#### 2.6.6 Hip pain with sit-to-stand

The 11-point numerical rating scale of pain (NRS-P 0–10) was used to assess hip pain during a movement known to be provocative for hip OA (sit-to-stand movement).^22^ Seat height was standardized to a set height (20 inches) and participants were provided standardized instructions to report the worst NRS-P from five repetitions. The NRS has excellent reliability in OA populations (*ICC* ​= ​0.95).^23^

#### 2.6.7 Lifestyle measures

Smoking status (Yes/No) and alcohol intake (drinks per week) were recorded.

#### 2.6.8 Medication usage

Participants listed all medications, which were then categorized as being for pain (Yes/No), hypertension (Yes/No), or hypercholesterolaemia (Yes/No).

#### 2.6.9 Pain location

Participants recorded areas of musculoskeletal aches, pains, and injuries beyond just hip/thigh pain (neck, shoulders, upper back, elbows, wrist/hands, lower back, knees, ankles/feet, none) in the previous 12 months using the Nordic Musculoskeletal Questionnaire.^24^^,^^25^

#### 2.6.10 Physical activity level

Participants self-reported (Yes/No) whether they performed moderate to vigorous physical activity most days of the week.

#### 2.6.11 Psychological measures

The generalized anxiety screen (GAD-7),^26^ patient health questionnaire (PHQ-9),^27^ and depression, anxiety, and stress scale (DASS)^28^ were completed by all participants.

### 2.7 Sample size estimation

Sample size calculations were performed using G.Power (v3.1.9.7.) for a fixed-effects analysis of variance. Between-group sample size was calculated using an estimated quadriceps voluntary activation effect size of (*f* ​= ​0.6), which is the lower limit of the 95% confidence interval for our systematic review quantifying voluntary activation deficits in knee OA. To detect a univariate between-group effect of 0.6 when *α* ​= ​0.05, power ​= ​0.90, a minimum sample of 32 participants was required (16 per group).

### 2.8 Statistical analysis

The distribution of data was examined visually and tested with the Kolmogorov-Smirnov Test for each variable. All data were recorded descriptively with between-group differences presented as mean (95%*CI*).

For our primary objective, we performed separate generalized linear models for each variable of interest (voluntary activation; SICI [RF]; ICF [RF]; SP [RF]) to evaluate between-group differences. All models were adjusted for age and sex (as these may influence voluntary activation). Other key variables (MVC, voluntary activation, HAGOS: Pain Subscale) were included on a model-by-model basis using a step-wise approach and excluded where the exclusion of variables improved the model's goodness-of-fit (i.e., indicated by Akaike information criterion improvement).

For our secondary objectives, generalized linear models were performed including all participants to: i) evaluate the association of the mean ICF, SICI and SP from the pooled RF, VL and VM (given all contribute to voluntary activation) with voluntary activation (adjusting for MVIC), ii) evaluate the association of the mean ICF, SICI and SP with hip-related pain (i.e., HAGOS Pain subscale score), and iii) evaluate the association of the mean ICF, SICI and SP with motor cortex excitability (i.e., RMT and AMT). Residuals of all generalized linear models were inspected. All data analysis was performed in IBM SPSS Statistics (v.29.0). Statistical significance was accepted when *p* ​< ​0.05.

#### 2.8.1 Sensitivity analysis

Sensitivity analysis was performed by individually adding the following variables to each model to assess for better model fit (via the Akaike's Information Criterion): ethnicity; smoking status; weekly alcohol intake; medications (pain); medications (hypertension); medications (hypercholesterolaemia); diagnosed mental health disorder; self-reported regular moderate to vigorous physical activity; history of knee pain (yes/no); GAD7; PHQ-9; DASS. If model fit was improved and this changed the significance of the primary variable of interest (i.e., group) this was reported.

## 3 Results

### 3.1 Participants

We included 41 participants: 17 with hip OA (76% female sex), and 24 without (92% female sex). This represents a larger number of control participants as we continued to recruit control participants until we reached the required sample for hip OA participants. Participants were a mean (standard deviation, *SD*) age of 58.7 (7.9) years and all participants were Australian residents, with complete demographic data by group provided (Appendix B).

### 3.2 Participant characteristics

Hip OA participants were slightly younger than our control participants (mean difference ​= ​5.1 years), had more regions of pain than control participants (mean difference ​= ​1.0 regions) and had more disability on their HAGOS subscales for: symptoms and stiffness; pain; physical function, daily living; function, sports, and recreational activities; and quality of life. There were no differences between groups for weekly alcohol intake. Hip OA participants had slightly higher, not-clinically significant scores for the GAD-7 (mean difference ​= ​1.6 points) and PHQ-9 (mean difference ​= ​1.7 points), but not the DASS (mean difference ​= ​2.5 points). Complete characteristics are presented in Table 2.

**Table 2:** Participant characteristics.

Variables	Controls (*n* ​= ​24)		Osteoarthritis (*n* ​= ​17)		Between group difference
	Mean (*SD*)	Median	Mean (*SD*)	Median	Mean (95% *CI*)
Age (years)	60.8 (5.7)	61.0 [21.0]	55.7 (9.6)	56.0 [38.0]	−5.1 (−9.9 to −0.2)
Alcohol Intake (standard drinks per week)	3.6 (3.4)	3.0 [12.0]	3.2 (3.7)	2.0 [12.0]	−0.5 (−2.7 to 1.8)
HAGOS: Symptoms and Stiffness (%)	4.3 (6.7)	0.0 [17.9]	30.0 (15.9)	28.6 [57.1]	25.7 (18.4–33.0)
HAGOS: Pain (%)	4.2 (8.4)	0.0 [35.0]	25.0 (15.0)	30.0 [45.0]	21.1 (13.6–28.5)
HAGOS: Physical function, daily living (%)	2.9 (8.3)	0.0 [40.0]	25.3 (18.7)	20.0 [55.0]	22.4 (13.7–31.1)
HAGOS: Function, sports and recreational activities (%)	4.3 (9.0)	0.0 [31.2]	36.9 (22.0)	32.2 [65.6]	32.6 (22.5–42.7)
HAGOS: Participation in physical activities (%)	31.3 (42.5)	0.0 [100.0]	46.2 (31.0)	37.5 [100.0]	11.4 (−13.1 to 35.9)
HAGOS: Quality of Life (%)	8.1 (13.8)	0.0 [40.0]	38.5 (23.3)	40.0 [85.0]	30.4 (18.7–42.1)
Hip pain with sit to stand (Total Score/10)	0.0 (0.0)	0.0 (0.0)	1.4 (1.9)	1.0 (5.0)	1.4 (0.7–2.2)
Number of pain regions (*n*)	1.6 (1.1)	1.0 [3.0]	2.6 (1.7)	2.0 [6.0]	1.1 (0.2–1.9)
GAD7 (Total Score/21)	0.7 (1.1)	0.0 [4.0]	2.3 (2.6)	2.0 [8.0]	1.6 (0.4–2.8)
PHQ9 (Total Score/27)	0.9 (0.9)	1.0 [3.0]	2.6 (3.1)	2.0 [12.0]	1.7 (0.4–3.1)
DASS (Total Score/63)	2.1 (2.2)	1.0 [9.0]	4.6 (4.7)	3.0 [15.0]	2.5 (0.2–4.7)

Legend: *n* ​= ​number, *SD* ​= ​standard deviation, *CI* ​= ​confidence interval, HAGOS= Hip and Groin Outcome Score with scored inverted so a higher score is worse and a lower score is better, % ​= ​percentage, GAD7 ​= ​7-item generalized anxiety screen, PHQ9 ​= ​9-item patient health questionnaire DASS ​= ​depression, anxiety and stress scale.

### 3.3 Voluntary activation

The mean (*SD*) for voluntary activation and quadriceps MVIC by group are presented (Table 3).

**Table 3:** Participant neurophysiological characteristics by group.

Variables	Control	Osteoarthritis
	Mean (*SD*), *n*	Mean (*SD*), *n*
**Quadriceps Voluntary Activation**		
Voluntary Activation	93.14 (4.89), 18	89.8 (6.57), 17
**Quadriceps Strength**		
Maximal Voluntary Isometric Contraction (N)	193.93 (43.97), 23	218.72 (70.71), 17
**Stimulator Intensities**		
Resting Motor Threshold (MSO)	66 (14),23	68 (14), 17
Active Motor Threshold (MSO)	47 (11), 23	46 (8), 17
**Corticospinal Excitability**		
Normalised Motor Evoked Potential: RF (% of Mmax)	16 (13), 21	20 (13), 21
Normalised Motor Evoked Potential: VL (% of Mmax)	16 (14), 21	18 (14), 21
Normalised Motor Evoked Potential: VM (% of Mmax)	14 (10), 21	20 (26), 21
**Intracortical Excitability**		
Intracortical facilitation: RF	1.02 (0.22), 21	1.14 (0.44), 15
Intracortical facilitation: VL	0.99 (0.22), 21	1.11 (0.41), 15
Intracortical facilitation: VM	1.05 (0.21), 21	1.09 (0.29), 15
Short-Interval Intracortical Inhibition: RF	0.71 (0.22), 21	0.66 (0.19), 15
Short-Interval Intracortical Inhibition: VL	0.72 (0.24), 21	0.62 (0.21), 15
Short-Interval Intracortical Inhibition: VM	0.75 (0.27), 21	0.61 (0.22), 15
Silent Period: RF (ms)	114 (50), 21	111 (45), 21
Silent Period: VL (ms)	115 (66), 21	109 (60), 21
Silent Period: VM (ms)	115 (56), 21	108 (60), 21

Legend: *n* ​= ​number, *SD* ​= ​standard deviation, N= Newtons, MSO= Percentage of Maximal Stimulator Output, VL= Vastus lateralis, RF= Rectus Femoris, VM= Vastus Medialis, % ​= ​percentage, MMax ​= ​Maximal M-wave, ms ​= ​milliseconds.

Note: Intracortical facilitation and short-interval intracortical inhibition are presented as a ratio of the motor evoked potential and reported to 2 decimal places – all other data are only reported without decimals as reported decimals would be smaller than what is able to be tested.

Our generalized linear model demonstrated that people with hip OA have less voluntary activation (*β* *=* -5.29, 95%*CI,* -0.79 to -9.79, *p* ​= ​0.021) compared to controls (Table 4, Appendix C). This model also demonstrated that less voluntary activation was associated with female sex, MVIC and increased pain (*β* *=* 0.16, 95%*CI,* 0.01 to 0.30, *p* ​= ​0.041, Table 4), while age had no effect. Sensitivity analyses did not change results.

**Table 4:** Generalized linear model of voluntary activation (Akaike's information criterion ​= ​215.9).

Variable	*β*	*SE*	95%*CI,*	*p*
Group (Control)	5.29	2.30	0.79 to 9.79	0.021
Age	0.17	0.12	−0.07 to 0.41	0.176
Sex (Female)	10.29	2.53	5.32 to 15.56	< 0.001
Maximal Voluntary Isometric Contraction	0.06	0.02	0.02 to 0.10	< 0.001
HAGOS: Pain	0.16	0.08	0.01 to 0.30	0.041
*Intercept*	*55.70*	*10.97*	*34.20to77.19*	*<0.001*

Legend: *β* ​= ​Beta-estimate, *SE* = Standard Error, % ​= ​Percentage, *CI* = Confidence Interval.

### 3.4 Corticospinal and intracortical responses

The mean (*SD*) for all transcranial magnetic stimulation outcomes by group are presented in Table 3.

#### 3.4.1 Resting and active motor threshold

Generalized linear models demonstrated that participants with hip OA did not differ in resting motor threshold (*β* *=* −4.76, 95%*CI*, −14.08 to 4.56, *p* ​= ​0.317; Appendix D) or active motor threshold (*β* *=* −2.13, 95%*CI,* −7.12 to 2.86, *p* ​= ​0.404; Appendix E) compared to controls. Sensitivity analyses did not change results.

#### 3.4.2 Intracortical facilitation

A generalized linear model demonstrated that participants with hip OA had greater ICF (*β* *=* 0.22, 95%*CI,* 0.01 to 0.43, *p* ​= ​0.044) compared to controls (Appendix F). Sensitivity analyses did not change results.

#### 3.4.3 Short-interval intracortical inhibition

A generalized linear model demonstrated that participants with hip OA had a similar level of SICI (*β* *=* −0.02, 95%*CI*, −0.15 to 0.006, *p* ​= ​0.806) compared to controls (Appendix G). Sensitivity analyses did not change results.

#### 3.4.4 Silent period

A generalized linear model demonstrated that participants with hip OA have a similar SP duration (*β* *=* 8.72, 95%*CI,* −24.75 to 42.20, *p* ​= ​0.610) compared to controls (Appendix H). Sensitivity analyses did not change results.

### 3.5 Association of intracortical facilitation and short-interval intracortical inhibition with voluntary activation

A generalized linear model (Table 5) demonstrated that greater voluntary activation was associated with a larger SICI ratio (*β* *=* 10.50, 95%*CI,* 2.00 to 18.99, *p* ​= ​0.015). Due to the nature of SICI being a ratio this means less inhibition was associated with a greater voluntary activation. Voluntary activation was not associated with ICF (*β* *=* 3.37, 95%*CI,* −3.15 to 9.89, *p* ​= ​0.311). Sensitivity analyses did not change results.

**Table 5:** Generalized linear model of voluntary activation- maximal (AIC ​= ​213.0).

Variables	*β*	*SE*	95%*CI*	*p*
Maximal Voluntary Isometric Contraction	0.02	0.02	−0.01 to 0.06	0.162
Intracortical facilitation: Quadriceps	3.37	3.33	−3.15 to 9.89	0.311
Short-Interval Intracortical Inhibition: Quadriceps	10.50	4.33	2.00 to 18.99	0.015
*Intercept*	*75.87*	*6.78*	*62.59to89.15*	*<0.001*

Legend: *β=* Beta-estimate, *SE* = Standard Error, % ​= ​Percentage, *CI* = Confidence Interval.

### 3.6 Association of motor cortex excitability to hip-related pain

#### 3.6.1 Resting motor threshold

Across groups, resting motor threshold was significantly associated with hip-related pain (HAGOS), with a 0.52% (*β* *=* 0.52, 95%*CI,* 0.18 to 0.86, *p* ​= ​0.003, Appendix I) higher motor threshold associated with a 1 ​% higher pain score (HAGOS).

#### 3.6.2 Active motor threshold

Across groups there no relationship between the active motor threshold and hip-related pain on the HAGOS (*β* *=* 0.52, 95%*CI,* −0.22 to 1.27, *p* *=* 0.166, Appendix J).

#### 3.6.3 Intracortical facilitation and short-interval intracortical inhibition

Across groups we demonstrated ICF was significantly associated with hip-related pain, with greater facilitation being associated with greater pain on the HAGOS (*β* *=* 24.55, 95%*CI,* 6.93 to 42.18, *p* *=* 0.006, Appendix K). There was no association between SICI and hip-related pain (*β* *=* −0.967, 95%*CI,* −24.372 to 22.439, *p* *=* 0.935, Appendix L).

#### 3.6.4 Silent period

Across groups, there was no relationship between the SP duration and hip-related pain on the HAGOS (*β* *=* −0.06, 95%*CI,* −0.16 to 0.05, *p* *=* 0.274, Appendix M).

### 3.7 Association of intracortical facilitation and short-interval intracortical inhibition with motor cortex excitability

#### 3.7.1 Resting motor threshold

Our generalized linear model demonstrated that the RMT was not significantly associated with either ICF or SICI (Appendix N).

#### 3.7.2 Active motor threshold

Our generalized linear model demonstrated that the AMT was significantly associated with both ICF and SICI, with an interaction effect also observed between ICF and SICI (Appendix O). Specifically, a higher AMT was associated with lower ratios for both ICF and SICI. This means that with a higher AMT (cortex becomes less excitable, requiring a higher intensity to activate) there is an associated reduction in facilitation and increase in inhibition.

## 4 Discussion

Our cross-sectional study of people living with hip OA demonstrated that they have lower quadriceps voluntary activation compared to controls, which may be driven in part by greater intracortical inhibition (but not facilitation). In contrast, people with hip OA demonstrated higher ICF than controls, and a greater level of ICF (but not inhibition) was associated with greater hip related pain (as measured via the HAGOS). Therefore, this research suggests hip OA alters the intracortical network excitability of the motor system.

Overall, participants with hip OA demonstrated poorer ability to activate quadriceps, however, the observed group deficit was not as large as that seen with knee OA.^4^ This may be due to the quadriceps not being a primary mover of the hip with only RF crossing the joint. Alternatively, 95% confidence intervals suggest the deficit compared to non-OA controls could be as large as 10%, which would be comparable to knee OA and may be seen with a larger sample size.

Further, more intracortical inhibition (SICI) was associated with lower levels of voluntary activation although SICI did not show group differences. Our research group reported a similar association has been reported after ACLR with TMS testing during weak contraction.^7^ By contrast, weaker intracortical inhibition and poor voluntary activation were described for people with chronic ACL lesions (most reconstructed) in a study that tested the leg muscles at rest.^29^

In addition, we demonstrated that increased HAGOS pain scores were associated with a reduction in voluntary activation. This is clinically relevant as those with higher levels of pain are likely to have a larger activation impairment and this can be factored into rehabilitation. We have previously hypothesized that poor voluntary activation may impair exercise capacity and hence, neuromuscular adaptation.^14^ Thus, the results of this study suggest people with hip OA may not adapt to resistance training interventions as optimally as non-OA controls. The impaired neural activation of the muscle and the reduced physiological strain that can be placed upon a muscle during rehabilitation may impair rehabilitation effectiveness.

Our findings point to a complex relationship between TMS-based measures and self-reported pain. We demonstrated lower excitability of the motor cortex (higher RMT), but not AMT, was associated with more self-reported hip-related pain (HAGOS). One explanation for this discrepancy may be that the motor cortical excitability of a resting muscle is influenced by all inputs to the motor cortex. One of these inputs could include an inhibitory influence of pain which could titrate motor cortex output with contraction to a set force level. For example, if the motor cortex is inhibited by pain at rest, then more voluntary excitatory drive would be required to generate the cortical output that makes the muscles contract to the target 10%. Hence, the resultant excitability of the cortex may be the same during the controlled contraction although there might be a different mix of inputs. Indeed, ICF measured during weak contraction differed between people with hip OA and controls, providing some evidence of altered cortical function despite the performance of the same task. One point of caution is that motor thresholds (e.g., RMT or AMT) are subject to many factors, such as scalp thickness or proximity of cortical neurons to the coil.

Previous research, including our own, has hypothesized that intracortical inhibition may be an underpinning contributor to motor system impairment in people with chronic pain conditions, with inhibitory changes also observed in both experimental muscle pain,^30^ and persistent knee OA.^9^ However, in OA these associations vary between studies with ICF having been associated with both increased,^6^ and decreased pain scores,^9^ even when the same outcome measure for pain has been used. Methodological differences in TMS between studies may explain these differences (e.g., assessment of ICF in the hand rather than the leg^6^ or assessment at rest^9^ vs. weak contraction). This does reflect the broader chronic pain literature, with little consistency observed.^31^ In our study, neither SICI nor SP were associated with pain. However, RMT and ICF (measured during weak contraction) were significantly associated with hip-related pain with higher RMT or ICF being associated with increased pain. Our findings support that further investigation of facilitation, and not just inhibition is warranted. For example, following, acute post-operative ACLR it is facilitation that appears to substantially change, as opposed to inhibition (Under Review). Previous research has also shown that ICF changes both during and after experimental pain, with changes in inhibition occurring after this.^8^ Thus, it is possible that increased facilitation is first observed as a response to pain, and that inhibition only increases as a secondary measure to maintain normative motor thresholds.

While ICF and SICI were not associated with RMT, both of these measures were associated with AMT. There was also a significant interaction between SICI and ICF, which would be expected. In our study, SICI and ICF, which reflect the operation of intracortical circuitry, were measured during weak voluntary contractions. As intracortical facilitatory and inhibitory neurons contribute to the excitability of motor cortical output cells, it is not surprising to find they are related to AMT which reflects cortical excitability during weak contraction. However, we also demonstrated that greater facilitation was associated with more pain, whereas greater inhibition was associated with less voluntary activation. As neither pain nor voluntary activation was measured during weak voluntary contraction, it suggests that the measured ICF and SICI are not solely a consequence of the level of voluntary activity but warrant investigation as neural mechanisms that may link pain perception to reduced voluntary activation.

The major clinical implication of this research is that people with hip OA have poorer quadriceps voluntary activation. This decrease in voluntary activation is likely to impair the capacity for neuromuscular adaptation with resistance training. ^14^ Thus, people with hip OA may not adapt to resistance training interventions as optimally as healthy populations and this should be considered when prescribing rehabilitation.

### 4.1 Strengths and limitations

The primary strength of this research was the conduct of neurophysiological assessment in accordance with accepted methods commonly employed in this field, detailed reporting of our sample and a comprehensive statistical analysis using modelled that was able to adjust for important covariates. Research exploring voluntary activation and TMS of the gluteal muscles would be optimal for hip OA, however, this is not currently possible as transcranial and electrical stimulation for EMG and force recording of the gluteal muscles would be far less accurate than the quadriceps. Thus, this novel study investigated the quadriceps, with all measures based on the RF, as it crosses the hip joint. Moreover, the quadriceps is an important functional muscle group and a target for exercise-based rehabilitation for people with hip OA. Our sample included predominantly females in both groups and a younger cohort in our hip OA sample. However, whilst our statistical approach, which adjusted for age and sex, statistically addressed these differences it would have been preferred to have a sample with identical demographics between groups. We did not perform between-group statistical comparisons for variables not directly related to our objectives as a large number of comparisons would make us at high risk of spurious results from type II error. However, we included 95% confidence intervals for between-group mean differences to facilitate interpretation. Finally, instead of performing a single generalized linear model for each aim, we performed separate generalized linear models for each outcome of interest of each aim, as this was the best approach for our sample size. However, whilst we reported the models with the best fit, if we had a larger sample size (> 100 participants based on a post-hoc power analysis), we would have been able to perform a single model with all covariates that could have reduced the likelihood of spurious findings.

## 5 Conclusion

This novel study demonstrated that people living with hip OA have impaired quadriceps function, specifically, they demonstrate poorer ability to voluntarily activate this muscle. This may have implications for resistance training, as they are physiologically limited in their ability to produce strong, voluntary muscle contractions. This study also demonstrates that motor cortex inhibition is significantly associated with voluntary activation, whilst motor cortex facilitation was significantly associated with pain intensity. Interventions that can modulate intracortical excitability and voluntary activation may be needed as an adjunct to improve outcomes from exercise rehabilitation for people with hip OA.

## Ethical approval statement

This research was reviewed and received approval by the Edith Cowan University Human Research Ethics Committee (2023-04080-MURPHY). The research was implemented in accordance with the Declaration of Helsinki. All participants provided informed, electronic consent.

## CRediT authorship contribution statement

**Myles C. Murphy:** Writing – review & editing, Writing – original draft, Supervision, Project administration, Methodology, Funding acquisition, Formal analysis, Data curation, Conceptualization. **Molly Coventry:** Writing – review & editing, Formal analysis, Data curation. **Janet L. Taylor:** Writing – review & editing, Supervision, Methodology, Funding acquisition, Data curation, Conceptualization. **Ebonie K. Rio:** Writing – review & editing, Methodology. **Andrea B. Mosler:** Writing – review & editing. **Jackie L. Whittaker:** Writing – review & editing. **Christopher Latella:** Writing – review & editing, Supervision, Data curation.

## Declaration of competing interest

None declared.
