# Supplementary material for: People with hip osteoarthritis have reduced quadriceps voluntary activation and altered motor cortex function
Source: Sports Med Health Sci. 2024 Sep 20;7(6):438–45. doi: 10.1016/j.smhs.2024.09.005 (PMC12766290; doi:10.1016/j.smhs.2024.09.005)
Supplement: Multimedia component 1 [file mmc1.docx]

**Appendix A. Exclusion Criteria for Transcranial Magnetic Stimulation**

| **Exclusion Criteria** |
| --- |
| Pregnancy |
| Neurological conditions/illness, including epilepsy/convulsion/seizure |
| Vascular, traumatic, tumoural, infectious, or metabolic lesion of the brain, even without history of seizure, and without anticonvulsant medication |
| Previous or current implants in their body that may be triggered or heated by an electrical current (e.g. pacemaker, intracranial shunts, artificial cochlea, etc) |
| Any mental implanted in their head (e.g. surgical clips, staples, shrapnel) |
| Frequent or intense headaches |
| Previous brain trauma or neurosurgical intervention |
| Serious medical complications (e.g. advanced pulmonary, cardiac, liver or kidney disease) |
| Currently taking neuropsychotropic drugs (e.g. antiepileptics, neuroleptics, benzodiazepines, antidepressants) or drugs with an effect on neuroplasticity (dopamine, fluoxetine or D-amphetamine, sodium or calcium channel blockers, NMDA receptor antagonists) |
| Sleep deprivation the night before |

**Appendix B. Categorical Participant Characteristics**

|  | | Control (n) | Osteoarthritis (n) |
| --- | --- | --- | --- |
| Sex | Female | 22 | 13 |
|  | Male | 2 | 4 |
| Gender | Woman | 22 | 13 |
|  | Man | 2 | 4 |
| Multi-lingual | No | 22 | 14 |
|  | Yes | 2 | 3 |
| Race | White | 23 | 16 |
|  | Asian | 1 | 0 |
|  | Aboriginal or Torres Strait Islander | 0 | 1 |
|  | Black | 0 | 1 |
| Smoking Status | No | 24 | 17 |
|  | Yes | 0 | 0 |
| Medications: Pain | No | 22 | 10 |
|  | Yes | 2 | 7 |
| Medications: Cholesterol | No | 17 | 14 |
|  | Yes | 7 | 3 |
| Medications: Hypertension | No | 15 | 16 |
|  | Yes | 9 | 1 |
| Mental Health Condition | No | 21 | 15 |
|  | Yes | 2 | 1 |
|  | Prefer not to say | 1 | 1 |
| Education Level | Less than high school degree | 3 | 0 |
|  | High school graduate (high school diploma or equivalent including GED) | 4 | 5 |
|  | Bachelor's degree | 10 | 4 |
|  | Master's degree | 3 | 5 |
|  | Doctoral degree | 4 | 3 |
| Household Income | Less than AUD $30,000 | 2 | 0 |
|  | Between AUD $30,000 - $49,999 | 1 | 2 |
|  | Between AUD $50,000 - $79,999 | 5 | 1 |
|  | Between AUD $80,000 - $99,999 | 2 | 0 |
|  | Between AUD $100,000 - $149,999 | 3 | 6 |
|  | Between AUD $150,000 - $199,999 | 5 | 5 |
|  | More than AUD $200,000 | 6 | 3 |
| Working Status | Working full-time | 5 | 10 |
|  | Working part-time | 8 | 6 |
|  | Retired | 10 | 1 |
|  | Other | 1 | 0 |
| Performs Moderate to Vigorous Physical Activity Most Days | No | 9 | 4 |
|  | Yes | 15 | 13 |

*Legend: n= number, GED= graduate entry diploma, AUD= Australian Dollars*

**Appendix C. Voluntary Activation**

**Figure. Box-plot displaying the distribution of maximal voluntary activation (%) by group**

**
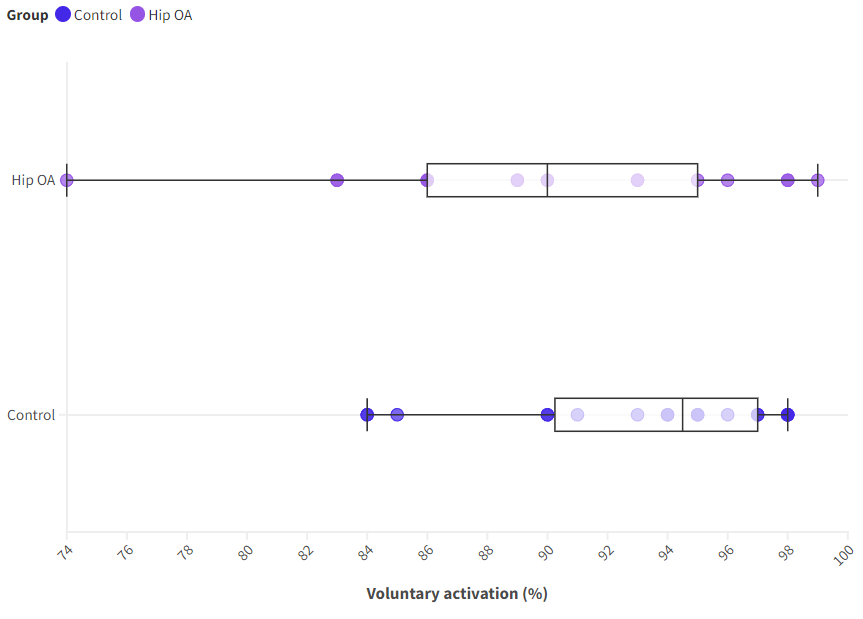
**

**Appendix D. Resting Motor Threshold**

**Table. Generalised Linear Model of Resting Motor Threshold** **(AIC=290.27)**

| **Variable** | **β** | **SE** | **95%CI** | **p** |
| --- | --- | --- | --- | --- |
| Group (Control) | -4.76 | 4.76 | -14.08 to 4.56 | 0.317 |
| Age | -0.17 | 0.30 | -0.75 to 0.41 | 0.567 |
| Sex (Female) | 4.13 | 7.60 | -10.77 to 19.03 | 0.587 |
| Voluntary Activation | 0.41 | 0.44 | -0.45 to 1.27 | 0.347 |
| *Intercept* | *37.07* | *41.66* | *-44.59 to 118.72* | *0.374* |

*Legend: β= Beta-estimate, SE= Standard Error, CI= Confidence Interval, %= Percentage*

**Figure. Box-plot displaying the distribution of resting motor threshold (Percentage of Maximal Stimulator Output) by group**

**
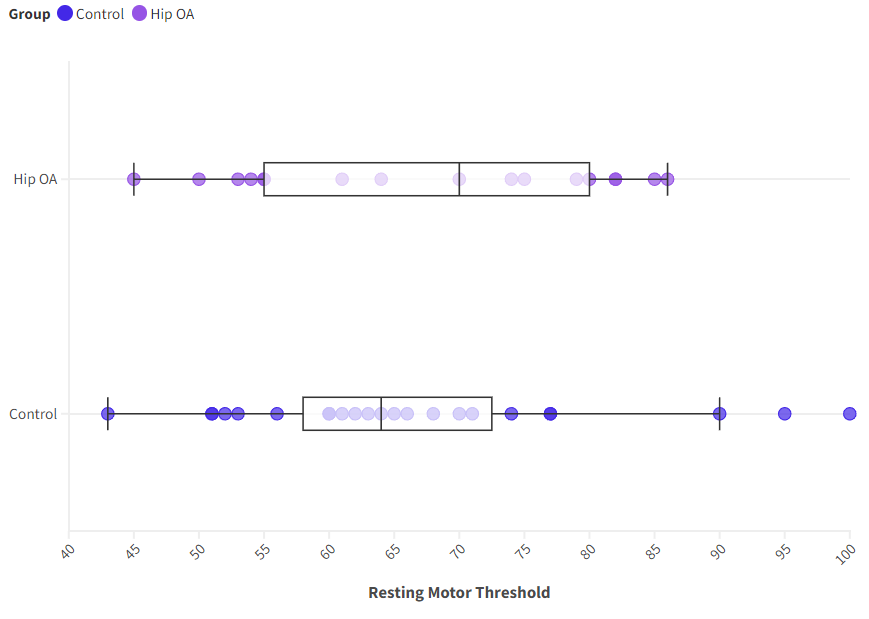
**

**Appendix E. Active Motor Threshold**

**Table. Generalised Linear Model of Active Motor Threshold** **(AIC=246.54)**

| **Variable** | **β** | **SE** | **95%CI** | **p** |
| --- | --- | --- | --- | --- |
| Group (Control) | -2.13 | 2.55 | -7.12 to 2.86 | 0.404 |
| Age | -0.042 | 0.16 | -0.35 to 0.27 | 0.793 |
| Sex (Female) | 4.40 | 4.07 | -3.57 to 12.38 | 0.279 |
| Voluntary Activation | 0.14 | 0.24 | -0.32 to 0.36 | 0.547 |
| *Intercept* | *31.84* | *22.30* | *-11.87 to 75.56* | *0.153* |

Legend: β= Beta-estimate, SE= Standard Error, CI= Confidence Interval, %= Percentage

**Figure. Box-plot displaying distribution of active motor threshold (Percentage of Maximal Stimulator Output) by group**


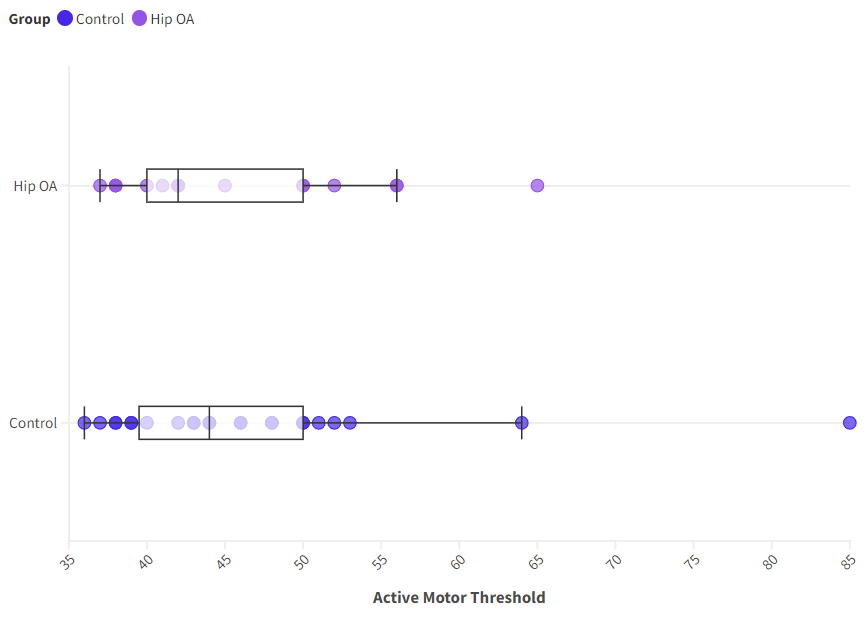


**Appendix F. Intracortical facilitation: Rectus Femoris**

**Table. Generalised Linear Model of Intracortical facilitation: Rectus Femoris** **(AIC=24.4)**

| **Variable** | **β** | **SE** | **95%CI** | **p** |
| --- | --- | --- | --- | --- |
| Group (Control) | -0.22 | 0.11 | -0.43 to -0.01 | 0.044 |
| Age | 0.021 | 0.01 | 0.01 to 0.04 | 0.005 |
| Sex (Female) | -0.06 | 0.18 | -0.41 to 0.29 | 0.758 |
| Voluntary Activation: Maximal | 0.01 | 0.01 | -0.01 to 0.03 | 0.269 |
| *Intercept* | *-1.008* | *1.00* | *-2.96 to 0.95* | *0.312* |

*Legend: β= Beta-estimate, SE= Standard Error, %= Percentage, CI= Confidence Interval*

**Figure. Box-plot displaying distribution of Intracortical facilitation: Rectus Femoris** **(Ratio of double pulse to single-pulse motor-evoked potential) by group**


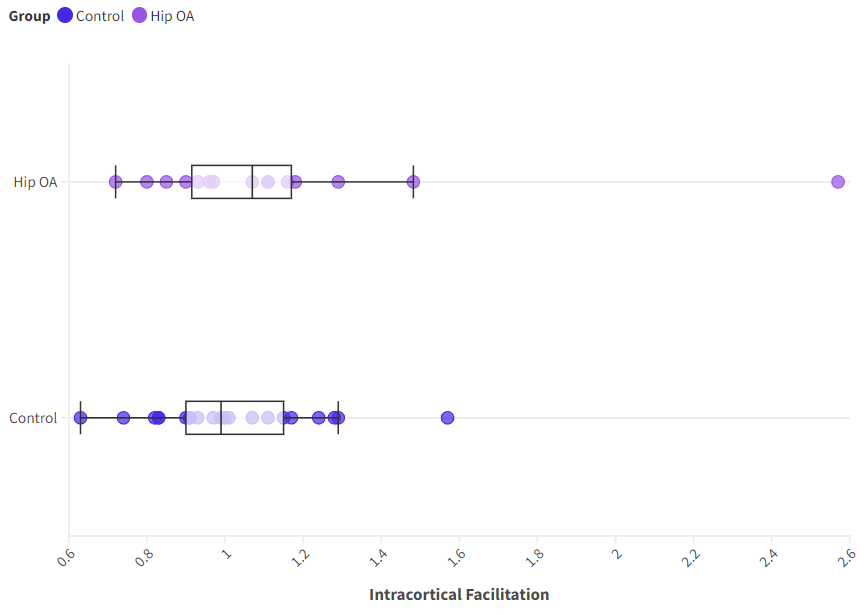


**Appendix G. Short-Interval Intracortical Inhibition: Rectus Femoris**

**Table. Generalised Linear Model of Short-Interval Intracortical Inhibition: Rectus Femoris** **(AIC= -8.5)**

| **Variable** | **β** | **SE** | **95%CI** | **p** |
| --- | --- | --- | --- | --- |
| Group (Control) | -0.02 | 0.07 | -0.15 to 0.06 | 0.806 |
| Age | 0.002 | 0.004 | -0.001 to 0.01 | 0.611 |
| Sex (Female) | 0.10 | 0.11 | -0.11 to 0.31 | 0.359 |
| Voluntary Activation: Maximal | 0.01 | 0.01 | -0.002 to 0.02 | 0.106 |
| *Intercept* | *-0.432* | *0.61* | *-1.62 to 0.75* | *0.475* |

*Legend: β= Beta-estimate, SE= Standard Error, %= Percentage, CI= Confidence Interval*

**Figure. Box-plot displaying distribution of Short-Interval Intracortical Inhibition: Rectus Femoris** **(Ratio of double pulse to single-pulse motor-evoked potential) by group**


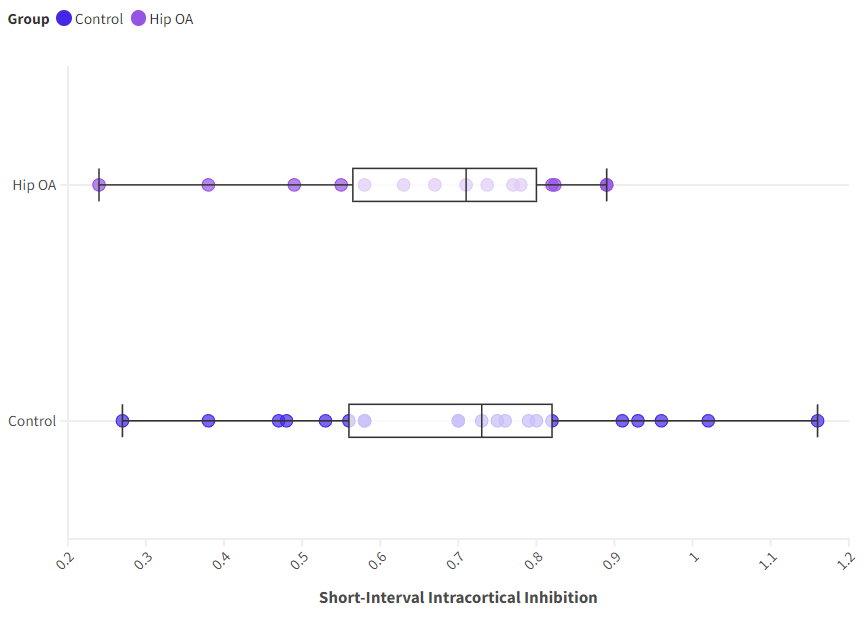


**Appendix H. Silent Period: Rectus Femoris**

**Table. Generalised Linear Model of Silent Period: Rectus Femoris (AIC= 368.1)**

| **Variable** | **β** | **SE** | **95%CI** | **p** |
| --- | --- | --- | --- | --- |
| Group (Control) | 8.72 | 17.08 | -24.75 to 42.20 | 0.610 |
| Age | 0.62 | 1.05 | -1.44 to 2.68 | 0.556 |
| Sex (Female) | -40.56 | 26.96 | -93.40 to 12.28 | 0.132 |
| Voluntary Activation: Maximal | 1.38 | 1.55 | -1.66 to 4.43 | 0.374 |
| *Intercept* | *-16.91* | *148.41* | *-307.79 to 273.96* | *0.909* |

*Legend: β= Beta-estimate, SE= Standard Error, %= Percentage, CI= Confidence Interval*

**Appendix I. Generalised Linear Model of the HAGOS Pain Subscale (AIC= 298.7)**

| **Variable** | **β** | **SE** | **95%CI** | **p** |
| --- | --- | --- | --- | --- |
| Age | -0.406 | 0.326 | -1.044 to 0.232 | 0.213 |
| Sex (Female) | -3.409 | 6.567 | -16.28 to 9.462 | 0.604 |
| Resting Motor Threshold | 0.519 | 0.174 | 0.177 to 0.861 | 0.003 |
| *Intercept* | *5.401* | *22.096* | *-37.907 to 48.709* | *0.807* |

Legend: β= Beta-estimate, SE= Standard Error, %= Percentage, CI= Confidence Interval

**Appendix J. Generalised Linear Model of the HAGOS Pain Subscale (AIC= 304.8)**

| **Variable** | **β** | **SE** | **95%CI** | **p** |
| --- | --- | --- | --- | --- |
| Age | -0.400 | 0.354 | -1.094 to 0.294 | 0.259 |
| Sex (Female) | -2.248 | 7.140 | -16.243 to 11.747 | 0.753 |
| Active Motor Threshold | 0.525 | 0.379 | -0.218 to 1.267 | 0.166 |
| *Intercept* | *15.582* | *26.70* | *-37.745 to 66.910* | *0.585* |

Legend: β= Beta-estimate, SE= Standard Error, %= Percentage, CI= Confidence Interval

**Appendix K. Generalised Linear Model of the HAGOS Pain Subscale (AIC= 299.9)**

| **Variable** | **β** | **SE** | **95%CI** | **p** |
| --- | --- | --- | --- | --- |
| Age | -0.807 | 0.362 | -1.516 to -0.098 | 0.026 |
| Sex (Female) | -1.542 | 6.630 | -14.523 to 11.452 | 0.816 |
| Intracortical Facilitation | 24.554 | 8.992 | 6.929 to 42.179 | 0.006 |
| *Intercept* | *35.481* | *19.630* | *-2.992 to 73.955* | *0.071* |

Legend: β= Beta-estimate, SE= Standard Error, %= Percentage, CI= Confidence Interval

**Appendix L. Generalised Linear Model of the HAGOS Pain Subscale (AIC= 306.7)**

| **Variable** | **β** | **SE** | **95%CI** | **p** |
| --- | --- | --- | --- | --- |
| Age | -0.407 | 0.364 | -1.120 to 0.305 | 0.262 |
| Sex (Female) | -0.961 | 7.606 | -15.869 to 13.947 | 0.899 |
| Short-Interval Intracortical Inhibition | -0.967 | 11.942 | -24.372 to 22.439 | 0.935 |
| *Intercept* | *37.995* | *22.692* | *-6.481 to 82.470* | *0.094* |

Legend: β= Beta-estimate, SE= Standard Error, %= Percentage, CI= Confidence Interval

**Appendix M. Generalised Linear Model of the HAGOS Pain Subscale (AIC= 314.8)**

| **Variable** | **β** | **SE** | **95%CI** | **p** |
| --- | --- | --- | --- | --- |
| Age | -0.54 | 0.32 | -1.18 to 0.09 | 0.094 |
| Sex (Female) | -3.14 | 7.12 | -17.09 to 10.81 | 0.659 |
| Silent Period | -0.06 | 0.05 | -0.16 to 0.05 | 0.274 |
| *Intercept* | *54.76* | *18.86* | *-17.79 to 91.73* | *0.004* |

Legend: β= Beta-estimate, SE= Standard Error, %= Percentage, CI= Confidence Interval

**Appendix N. Generalised Linear Model of the Resting Motor Threshold (AIC=277.0)**

| **Variable** | **β** | **SE** | **95%CI** | **p** |
| --- | --- | --- | --- | --- |
| Intracortical Facilitation: Rectus Femoris | -33.916 | 28.477 | -89.731 to 21.899 | 0.234 |
| Short-Interval Intracortical Inhibition: Rectus Femoris | -37.481 | 38.979 | -113.879 to 38.916 | 0.336 |
| (Intracortical Facilitation: Rectus Femoris) x (Short-Interval Intracortical Inhibition: Rectus Femoris) | 61.854 | 38.975 | -14.535 to 138.243 | 0.113 |
| HAGOS Pain Subscale | 0.345 | 0.111 | 0.127 to 0.562 | 0.002 |
| *Intercept* | *77.204* | *28.108* | *22.113 to 132.295* | *0.006* |

Legend: β= Beta-estimate, SE= Standard Error, %= Percentage, CI= Confidence Interval

**Appendix O. Generalised Linear Model of the Active Motor Threshold (AIC=222.6)**

| **Variable** | **β** | **SE** | **95%CI** | **p** |
| --- | --- | --- | --- | --- |
| Intracortical Facilitation: Rectus Femoris | -29.353 | 13.588 | -55.984 to -2.721 | 0.031 |
| Short-Interval Intracortical Inhibition: Rectus Femoris | -49.210 | 18.768 | -85.995 to -12.425 | 0.009 |
| (Intracortical Facilitation: Rectus Femoris) x (Short-Interval Intracortical Inhibition: Rectus Femoris) | 56.150 | 18.720 | 19.459 to 92.841 | 0.003 |
| *Intercept* | *68.143* | *13.526* | *41.632 to 94.654* | *<0.001* |

Legend: β= Beta-estimate, SE= Standard Error, %= Percentage, CI= Confidence Interval
